# [Retracted] Effects of Grb2‑associated binding protein 2‑specific siRNA on the migration and invasion of MG‑63 osteosarcoma cells

**DOI:** 10.3892/ol.2024.14497

**Published:** 2024-06-10

**Authors:** Huan Wang, Hui He, Hongmei Meng, Yang Cui, Wenbo Wang

Oncol Lett 15: 926–930, 2018; DOI: 10.3892/ol.2017.7375

Following the publication of this paper, it was drawn to the Editor's attention by a concerned reader that certain of the cell invasion assay data shown in Fig. 4A on p. 928 were strikingly similar to data that had appeared in different form in different articles written by different authors that had already been published.

Owing to the fact that the contentious data in the above article were already been published prior to its submission to *Oncology Letters*, the Editor has decided that this paper should be retracted from the Journal. The authors were asked for an explanation to account for these concerns, but the Editorial Office did not receive a satisfactory reply. The Editor apologizes to the readership for any inconvenience caused.

